# Effect of levosimendan treatment in cardiac surgery: a network meta-analysis of randomized controlled trials

**DOI:** 10.3389/fcvm.2025.1673410

**Published:** 2026-02-11

**Authors:** Binlu Zhu, Wanling Zhao, Yifei Li

**Affiliations:** Department of Pediatrics, West China Second University Hospital, Sichuan University, Chengdu, Sichuan, China

**Keywords:** cardiac surgery, heart failure, levosimendan, network meta-analysis, randomized controlled trials

## Abstract

**Background:**

The prophylactic administration of levosimendan in patients undergoing cardiac surgery remains clinically contentious, particularly regarding its efficacy and safety in improving key postoperative outcomes, such as cardiac function, renal protection, and mortality in high-risk surgical populations. Existing randomized controlled trials (RCTs) are heterogeneous in sample size, timing, and dosing of levosimendan and comparator inotropes and have yielded neutral or conflicting results for these endpoints. Moreover, prior meta-analyses have predominantly used pairwise comparisons and have not systematically compared levosimendan with all major inotropic alternatives (e.g., dobutamine, milrinone, and standard therapy) across multiple clinically relevant outcomes. As a result, the relative benefits and harms of levosimendan vs. other perioperative inotropic strategies in cardiac surgery remain unclear. To evaluate the potential benefits and risks of perioperative levosimendan therapy, we conducted a systematic network meta-analysis of available evidence.

**Methods:**

A comprehensive literature search was conducted in PubMed, Embase, the Cochrane Library, and other databases for RCTs evaluating perioperative levosimendan vs. placebo or alternative inotropic therapies, published up to 31 December 2024. Primary outcomes included cardiac index, central venous pressure (CVP), mean arterial pressure (MAP), intensive care unit (ICU) length of stay, and creatinine levels. The frequentist surface under the cumulative ranking curve (SUCRA) was calculated for each outcome to rank competing interventions.

**Results:**

A total of 29 RCTs encompassing 4,509 patients were included. Levosimendan was associated with higher postoperative CI [standardized mean difference (SMD) 1.16, 95% CI 0.04–2.29] compared with placebo and lower postoperative MAP (SMD −0.93, 95% CI −1.62 to −0.23) compared with dobutamine. Milrinone had lower CVP compared with dobutamine (SMD −0.66, 95% CI −1.22 to −0.10) and placebo (SMD −0.46, 95% CI −0.83 to −0.09), and levosimendan (SMD 0.40, 95% CI 0.05–0.75) had higher CVP compared with milrinone. Both levosimendan (SMD −0.68, 95% CI −1.13 to −0.24) and milrinone (SMD −0.71, 95% CI −1.25 to −0.17) significantly shortened ICU stays compared with dobutamine. No significant differences in creatinine levels were identified across interventions in the network meta-analysis.

**Conclusion:**

Levosimendan improved postoperative hemodynamic parameters, showing a higher cardiac index than placebo and shorter ICU stays than dobutamine, but it did not provide significant renal protection as assessed by creatinine levels.

**Systematic Review Registration:**

https://www.crd.york.ac.uk/PROSPERO/recorddashboard, identifier CRD42024612151.

## 1 Introduction

Levosimendan is a calcium sensitizer that enhances the binding of calcium ions to cardiac troponin by interacting with the troponin C subunit on cardiomyocytes, thereby promoting myocardial contractility and improving hemodynamic parameters in patients with heart failure (HF) (1). It increases myocyte contractility without increasing oxygen demand while simultaneously improving diastolic function and stimulating sympathetic nervous system activity (2). Additionally, it exerts vasodilatory effects and mildly inhibits phosphodiesterase (PDE) through the activation of adenosine triphosphate (ATP)-sensitive potassium channels. Its positive inotropic effect is independent of adrenergic stimulation and can be used in patients undergoing β-blocker therapy (3, 4).

Previous studies have mainly focused on patients with established heart failure or other cardiac conditions, leaving the potential benefits of levosimendan in the setting of cardiac surgery less well-defined. In recent years, levosimendan has been compared with other inotropic agents such as dobutamine, milrinone, noradrenaline, and adrenaline (5–8). Milrinone is commonly used to prevent postoperative low cardiac output syndrome (LCOS) and related symptoms, yet its use may be associated with adverse effects including tachycardia, increased myocardial oxygen consumption, and even myocardial necrosis (9). Several studies have demonstrated that perioperative levosimendan administration may improve postoperative outcomes following cardiac surgery. Furthermore, compared with placebo (10), dobutamine (11) or milrinone (12), levosimendan has been associated with higher rates of successful weaning from cardiopulmonary bypass, lower incidence of periprocedural myocardial infarction, and reduced lactate levels due to its favorable effects on tissue perfusion.

Despite these promising findings, large-scale comparative trials have not consistently confirmed the clinical benefits of levosimendan (13–15). Recently, several large multicenter randomized controlled trials (RCTs) on perioperative levosimendan in cardiac surgery have reported no significant improvement in postoperative clinical outcomes (16–18). To date, no systematic review has demonstrated the superiority of any inotropic drug over others in terms of major clinical endpoints. Therefore, we conducted a network meta-analysis to comprehensively evaluate the advantages and disadvantages of levosimendan in the perioperative management of cardiac surgery.

## 2 Methods

### 2.1 Study protocol

This systematic review was conducted in accordance with the Preferred Reporting Items for Systematic Reviews and Meta-Analyses (PRISMA) guidelines. The study protocol was prospectively registered with PROSPERO (Registration number: CRD42024612151).

### 2.2 Search strategy

A comprehensive systematic search was performed in PubMed, the Cochrane Library, and Embase databases for studies published prior to 31 December 2024. The search strategy incorporated the keywords “levosimendan” and “cardiac surgery,” as well as relevant Medical Subject Headings (MeSH) terms and associated keywords. The entry terms included “levosimendan” or “simendan” and “cardiac surgery” or “thoracic surgery” or “heart surgery.” All randomized controlled trials evaluating the administration of levosimendan in cardiac surgery patients were considered. There were no restrictions on control drugs or study design in the search terms, and no language restrictions were applied at the search stage. However, during study selection, we restricted inclusion to full-text articles published in English. Similar strategies were applied to other databases. All RCTs evaluating the administration of levosimendan in cardiac surgery patients were considered.

### 2.3 Study selection

Two reviewers (YL and BZ) independently screened the titles and abstracts obtained from the database searches. Potentially eligible studies were then subjected to full-text review by two reviewers (BZ, WZ) for inclusion based on predetermined criteria. Significant disagreements were resolved through discussion. To ensure comprehensive coverage, manual searches of reference lists from included articles and relevant review papers were conducted, and forward citation tracking of included studies was performed using Google Scholar.

Our study inclusion criteria were as follows according to the PICOS framework: (1) population: cardiac surgery patients, without limitation of age and type of cardiac surgery; (2) intervention: levosimendan; (3) comparison intervention: any control (placebo, standard inotropic therapies, dobutamine, milrinone); (4) outcome: cardiac index, central venous pressure (CVP), mean arterial pressure (MAP), intensive care unit (ICU) stays and creatinine; (5) study design: RCTs. We used the following criteria for study exclusion: (1) review, case report, or abstract; (2) animal or cell studies; (3) studies lacking a relevant outcome; (4) articles not published in English.

### 2.4 Data collection and assessment of study quality

Two independent investigators (BZ, WZ) performed data extraction and quality assessment of eligible studies. Any discrepancies in data interpretation were resolved through consensus discussion, with arbitration by a third reviewer when necessary. The quality of evidence for each outcome was systematically evaluated using the GRADE methodology (19, 20).

The following data were collected from each study: first author name, area, publication date, study design, number of patients, comparison intervention, and primary outcome (CI, CVP, MAP, ICU stays, and creatinine). When means and standard deviations were not reported, these values were estimated from medians, interquartile ranges, and sample sizes (21, 22). The baseline characteristics of included studies are presented in Table 1.

**Table 1:** Basic characteristics of included studies.

Author	Area	Year	Study design	Study population	LEVO			Control			Continuous infusion dose	Control	Continuous infusion dose	Assessment
					n	male	age	n	male	age				
Wang, A.	China	2019	A single-center, double-blind, randomized, placebo-controlled trial	≤48 months old and undergo cardiac surgery	94	55	5 (2, 11) months	93	52	7 (2,16) months	0.05 μg/kg/min for 48 h	Placebo	–	CI, Cr, ICU stay
Landoni, G.	Italy, Russia, Brazil, Australia	2017	A multicenter, randomized, double-blind, placebo-controlled trial	Perioperative hemodynamic support was indicated after cardiac surgery	248	159	66 (58, 74) years	258	168	66 (58,72) years	0.05 μg/kg/min (0.025–0.2 μg/kg/min) up to 48 h	Placebo	–	CI, Cr, CVP, ICU stay, MAP
Baysal, A.	Turkey	2014	A prospective, double-blinded, randomized clinical trial	low ejection fraction undergoing mitral valve surgery	64	22	56.7 ± 11.7 years	64	39	58.4 ± 9.8 years	0.1 μg/kg/min for 24 h	Standard inotropic therapies	–	CI, Cr, CVP, MAP, ICU stay
van Diepen, S.	Canada	2020	A double-blind, multicenter, randomized controlled trial	CABG	283	243	65 (59, 72) years	280	227	64 (58, 71) years	for 24 h	Placebo	–	CI, ICU stay
				Isolated valve surgery	49	24	65 (58, 73) years	48	25	71 (64, 76) years				
				Combined CABG and valve	96	80	67 (60, 75) years	92	78	65 (56, 73) years				
Bragadottir, G.	Sweden	2013	A prospective, placebo-controlled, and randomized trial	mechanically ventilated postcardiac surgery	15	14	65.5 ± 3.3 years	15	14	69.3 ± 2.7 years	0.1 µg/kg/min for 30 min urine collection periods	Placebo	–	CI, CVP, MAP
Anastasiadis, K.	Greece	2016	A prospective, double-blind, randomized study	CABG with reduced LVEF (≤40%)	16	14	61.1 ± 9.4 years	16	16	62.2 ± 11 years	0.1 μg/kg/min for 24 h	Placebo	–	CI
Shah, B.	India	2014	A prospective, double-blind, randomized study	OPCAB with low LVEF (<30%)	25	15	59.9 ± 8.8 years	25	16	61.3 ± 7.6 years	200 μg/kg for 24 h	Placebo	–	CI, CVP, MAP
Erb, J.	Germany	2014	A double-blind, single-center, prospective, randomized, placebo-controlled trial	>18 years with CABG surgery with LVEF ≤ 30%	17	13	69.5 ± 11.5 years	16	15	63.4 ± 7.8 years	0.1 µg/kg/min	Placebo	–	CI, CVP, ICU stay, MAP
Zangrillo, A.	Italy	2020	A multicenter, randomized, double-blind, placebo-controlled trial	Cardiac surgery	248		66 (58, 74) years	258		66 (58,72) years	0.05 μg/kg/min for up to 48 h or until ICU discharge	Placebo	–	CI, CVP, MAP
Alvarez, J.	Spain	2013	A sub-study of a randomized controlled study	Low cardiac output state after cardiac surgery	12	5	71.5 ± 5.2 years	13	6	72.7 ± 5.0 years	0.2 μg/kg/min for 24 h	Dobutamine	7.5 μg/kg/min for 24 h	CI, CVP, MAP
Kandasamy, A.	India	2017	A prospective, randomized, double-blind study	OPCAB	40	31	55.2 ± 3.2 years	40	33	54.8 ± 3.7 years	0.1 μg/kg/min for 2 h	Dobutamine	5 μg/kg/min for 24 h	CI, ICU stay, MAP
Ebade, A. A.	Kingdom of Saudi Arabia	2013	A prospective randomized study	PAP for children undergoing cardiac surgery repair of cardiac septal defects	25	16	19.8 ± 9.9 months	25	13	17.5 ± 7.8 months	0.1–0.2 μg/kg/min	Dobutamine	4–10 μg/kg/min	CI, ICU stay
Guerrero Orriach, J. L.	Spain	2020	A double-blind clinical trial	LCOS after cardiac surgery	50	29		50	38		0.1 μg/kg/min	Dobutamine	5 μg/kg/min	CI, Cr, CVP, ICU stay, MAP
Jothinath, K.	India	2021	A single-center, randomized study	Infants undergoing corrective surgery for congenital heart disease	20		6.85 ± 3.58 months	20		6.85 ± 3.57 months	0.1 μg/kg/min	Milrinone	0.5 μg/kg/min	CI, ICU stay
Thorlacius, E. M.	Swedish	2021	A *post hoc*, no prespecified exploratory secondary analysis from a randomized, prospective, double-blinded clinical trial	Infants 1–12 months old, diagnosed with VSD, complete atrioventricular septal defect, or tetralogy of Fallot undergoing corrective surgery	32	16	5.9 ± 2.9 months	38	18	5.6 ± 2.7 months	–	Milrinone	–	CI, CVP, MAP, ICU stay,
Fredholm, M.	Sweden	2018	A prospective, randomized, blinded, double-armed study	Patients after aortic valve replacement for aortic stenosis	15	9	70 ± 9.3 years	16	11	71 ± 8.2 years	0.1–0.2 μg/kg/min	Milrinone	0.4–0.8 μg/kg/min	CI, CVP, MAP
Abril-Molina, A.	Spain	2021	A randomized, placebo-controlled, double-blinded study	Patients were aged between 28 days and 13 years, and were scheduled	15	9	0.6 (0.5, 4) years	15	10	0.9 (0.6,8) years	0.2 µg/kg/min	Placebo	–	Cr, CVP, ICU stay, MAP
Zangrillo, A.	Italy	2018	A *post hoc* analysis of a multicenter, randomized, double-blind, placebo-controlled trial	Patients undergoing mitral valve surgery	46	20	68 (60, 76) years	44	20	68 (63,78) years	starting dose of 0.05 μg/kg/min, 0.025–0.2 μg/kg/min for up to 48h	Placebo	–	Cr
De Hert, S. G.	Belgium	2007	A single-center, randomized study	Patients with a preoperative ejection fraction 30% scheduled for elective cardiac surgery	15	5	67 ± 11 years	15	5	69 ± 10 years	0.1 µg/kg/min	Milrinone	0.5 μg/kg/min	Cr, CVP, ICU stay, MAP
Momeni, M.	Belgium	2011	A prospective, randomized, double-blind clinical study	Patients between 0 and 5 years old requiring inotropic support for corrective congenital heart surgery	18		109 (8, 953) days	18		145 (7,977) days	0.05 μg/kg/min	Milrinone	0.4 μg/kg/min	Cr, ICU stay, MAP
Tholén, M.	Sweden	2021	A prospective, placebo-controlled, and randomized trial	Postcardiac surgery patients	16	10	70 (52, 82)	13	12	65 (37,78)	0.1 µg/kg/min	Placebo	–	CVP, MAP
Lechner, E.	Austria	2012	A prospective, single-center, double-blind, randomized pilot study	Infants undergoing corrective open-heart surgery	20	11	78.4 ± 80.5 days	20	11	63.9 ± 81.3 days	0.1 µg/kg/min	Milrinone	0.5 μg/kg/min	ICU stay
Ricci, Z.	Italy	2012	A prospective randomized open-label study	Neonates undergoing RACHS 3 and 4	32	20	18.7 ± 14 days	31	17	15.5 ± 9.2 days	0.1 µg/kg/min for 72 h	Standard inotropic therapies	–	ICU stay, MAP
Mehta, R. H.	United States, Canada, Austria, Germany	2017	A multicenter, randomized, placebo-controlled, phase 3 trial	patients with a left ventricular ejection fraction of 35% or less who were undergoing cardiac surgery	428	347	65 (59, 73) years	421	332	65 (58,72) years	0.2 µg/kg/min for 1 h, followed by 0.1 µg/kg/min for 23 h	Placebo	–	ICU stay
Leppikangas, H.	Finland	2011	A prospective randomized study	LVEF <50% or LV hypertrophy	12	10	76 ± 10 years	12	8	75 ± 8 years	0.2 µg/kg/min for 24 h	Placebo	–	ICU stay
Cholley, B.	France	2017	A randomized, double-blind, placebo-controlled trial	Patients with a LVEF ≤ 40% and scheduled for isolated or combined CABG	167	139	69 ± 10 years	168	143	67 ± 10 years	0.1 µg/kg/min for 24 h	Placebo	–	ICU stay
Tritapepe, L.	Italy	2009	A double-blind, single-center, prospective, randomized, placebo-controlled trial	Age ≥18 years and an intention to perform first-time multivessel CABG	52	43	64 (54, 86) years	50	39	66.5 (54,87) years	0.1 µg/kg/min for 24 h	Placebo	–	ICU stay
Nag, P.	India	2023	A prospective, randomized, controlled trial	Children between 1 month and 12 years presenting with VSD and PAH	66	25	25.2 ± 34.6 years	66	27	26.7 ± 34.5 years	0.1–0.15 µg/kg/min for 48 h	Milrinone	0.5–1 μg/kg/min	ICU stay, MAP
Mishra, A.	India	2016	A prospective, randomized study	Patients with valvular heart disease and PAH undergoing valve surgery	20		37.3 ± 11.6 years	20		43.7 ± 13.1 years	0.1 µg/kg/min for 24 h	Milrinone	0.5 μg/kg/min	ICU stay

LEVO, levosimendan; CI, cardiac index; CVP, central venous pressure; MAP, mean systemic artery pressure; ICU, intensive care unit; CABG, coronary artery bypass grafting; LVEF, left ventricular ejection fraction; OPCAB, off-pump coronary artery bypass grafting; PAP, pulmonary artery pressure; LCOS, low cardiac output syndrome; VSD, ventricular septal defects; PAH, pulmonary artery hypertension; RACHS, risk-adjusted classification for congenital heart surgery.

The risk of bias in the included RCTs was assessed using the original Cochrane risk of bias tool, according to the Cochrane Handbook for Systematic Reviews of Interventions (version 5.1.0) (23). The following domains were evaluated: random sequence generation (selection bias), allocation concealment (selection bias), blinding of participants and personnel (performance bias), blinding of outcome assessment (detection bias), incomplete outcome data (attrition bias), selective reporting (reporting bias), and other bias (24).

### 2.5 Statistical analysis

Meta-analyses were conducted to synthesize the effects of each outcome. Binary outcomes were analyzed using odds ratios (ORs) with 95% confidence intervals (CIs), while continuous outcomes were evaluated through standardized mean differences (SMDs) with corresponding 95% CIs, both derived from random-effects models to account for potential heterogeneity among studies. Publication bias was evaluated through visual inspection of funnel plots and quantified using Egger's linear regression test (25). A two-sided *P*-value < 0.05 was considered statistically significant.

Network meta-analysis was performed using the hierarchical summary receiver operating characteristic (HSROC) model to estimate and compare SROC curves (25). Treatment rankings were quantified through frequentist surface under the cumulative ranking curve (SUCRA) values for each outcome (26). These values, ranging from 0% (indicating the lowest-ranked, least effective treatment) to 100% (representing the highest-ranked, most effective treatment), provided a quantitative measure of comparative treatment efficacy.

All statistical analyses were performed using Stata version 18.0 (27), employing frequentist multivariate meta-analysis (commands network meta and mvmeta) for network evaluation, supplemented by publication bias and sensitivity analyses.

## 3 Results

### 3.1 Included studies

**Figure 1:**
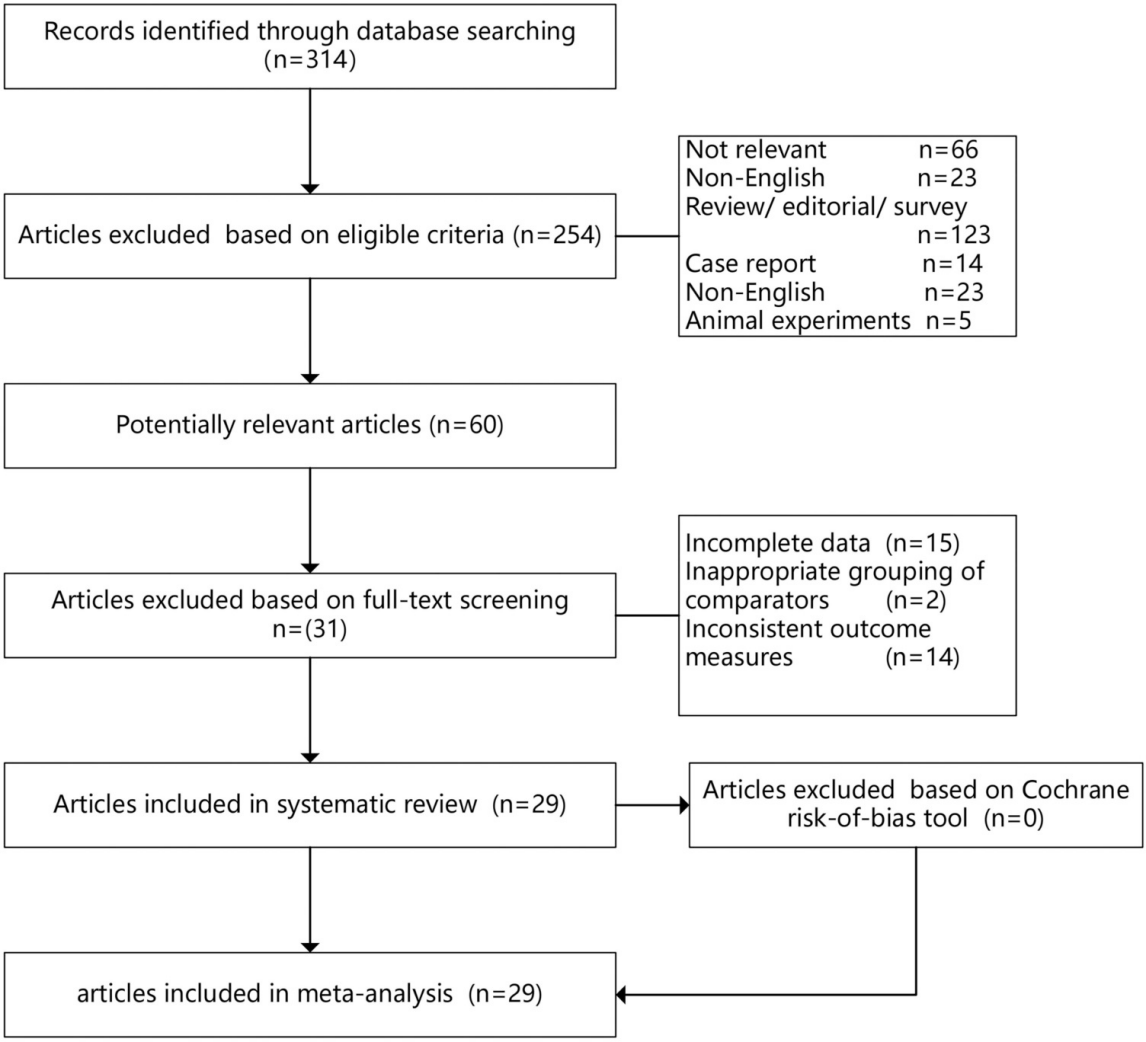
Preferred Reporting Items for Systematic Reviews and Meta-Analyses (PRISMA) flow diagram of randomized controlled trials included and excluded.

A total of 462 articles were initially identified through database searches. After removing 148 duplicates, 314 articles remained. Subsequently, 254 articles were excluded based on eligibility criteria, including irrelevance (*n* = 66), non-English language (*n* = 23), review/editorial/survey articles (*n* = 146), case reports (*n* = 14), and animal experiments (*n* = 5). Following initial screening, 60 potentially relevant articles were assessed in detail. Of these, 31 articles were excluded after full-text review due to incomplete data (*n* = 15), inappropriate grouping of comparators (*n* = 2), or inconsistent outcome measures (*n* = 14). Ultimately, 29 studies involving a cumulative cohort of 4,509 participants were incorporated into our analysis (Figure 1). No additional studies were identified through reference tracking. The baseline characteristics of included studies are presented in Table 1. Among these, five were multicenter trials, and twenty-four were single-center trials. In terms of control agents, fifteen studies used a placebo (10, 16, 28–33), eight used milrinone (8, 12, 34–39), four used dobutamine (6, 7, 40, 41), and two used standard therapy (5, 42). Sixteen trials reported postoperative CI, thirteen reported CVP, seventeen reported MAP, twenty-one reported ICU stays, and eight reported creatinine.

### 3.2 Study quality and publication bias

**Figure 2:**
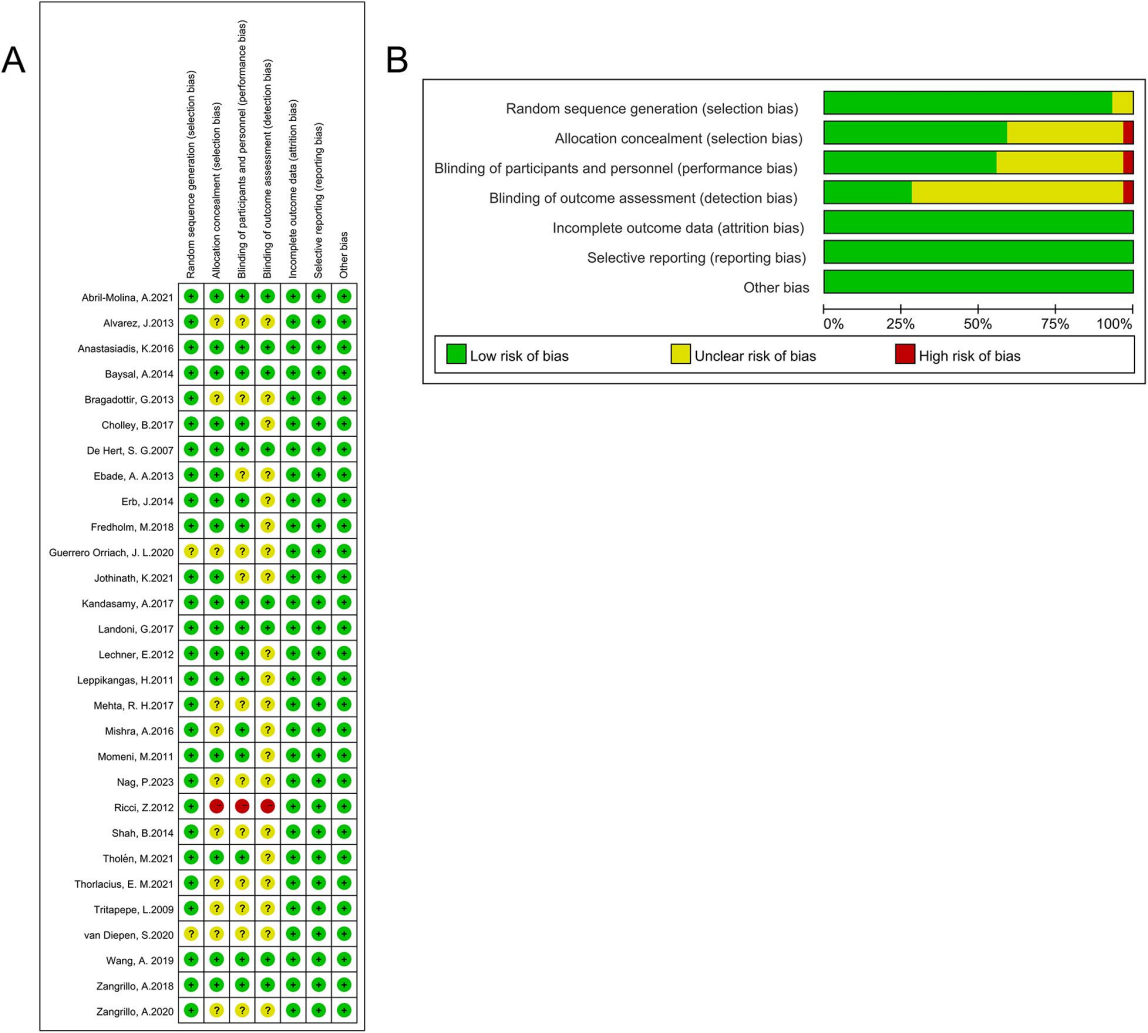
Cochrane Collaboration's risk-of-bias tool assessment of the quality of a randomized controlled trial (RCT). Risk of bias summary: The judgements about each risk of bias item for each included study **(A)**. Risk of bias graph: The judgements about each risk of bias item reflected s percentages across all included studies **(B)**.

The Cochrane risk of bias tool was employed to evaluate the quality of the included studies. Most of the studies demonstrated moderate to high quality, with eight studies (27.6%) rated as low risk of bias and the remaining studies (72.4%) classified as moderate risk of bias. The most common bias was detection bias. Detailed results of the risk of bias are presented in Figure 2.

### 3.3 Network meta-analysis

#### 3.3.1 Cardiac index

**Figure 3:**
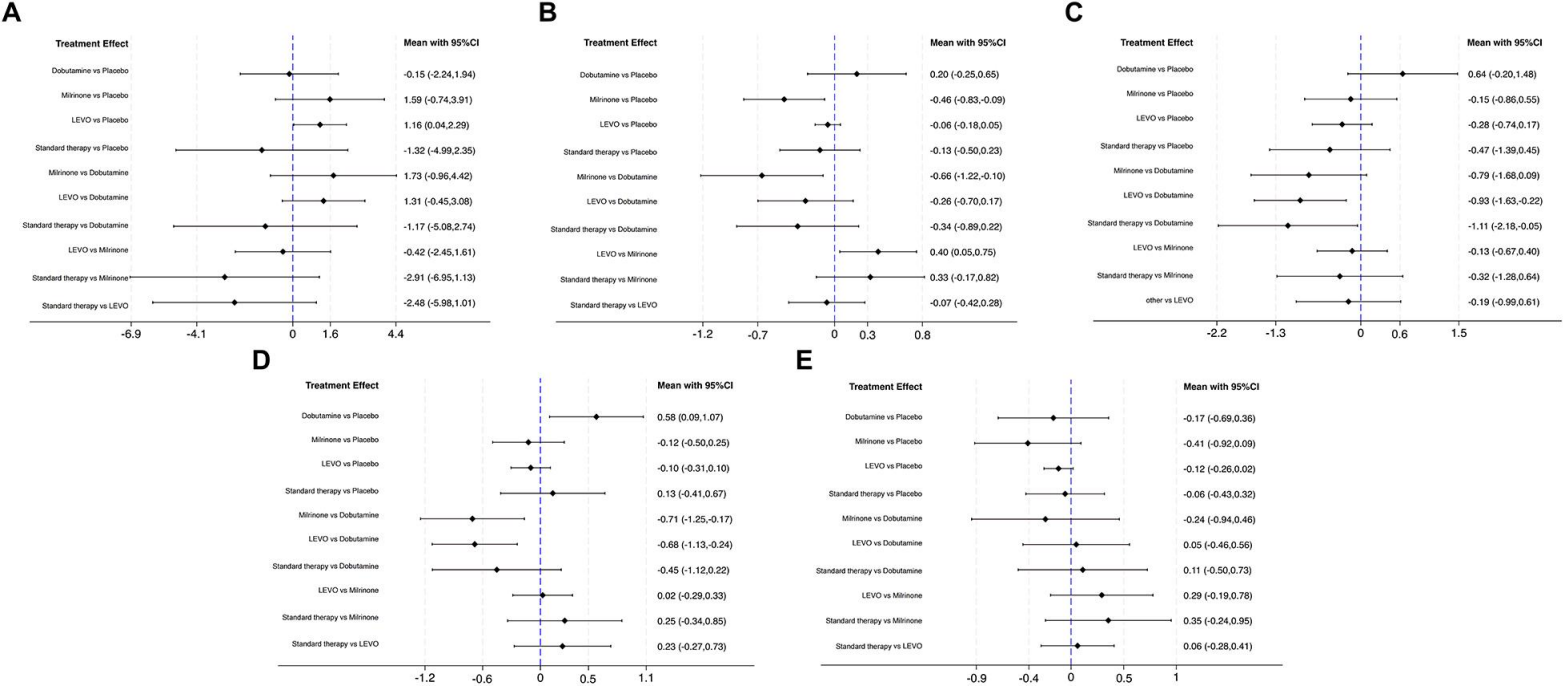
Forest plot of network meta-analysis for CI **(A)**, CVP **(B)**, MAP **(C)**, ICU stays **(D)**, and creatinine **(E)**.

Sixteen RCTs systematically compared the effects of levosimendan vs. alternative therapies on CI (5–8, 10, 16, 28–33, 35, 36, 40, 41) (Supplementary Figure S1A). The network meta-analysis demonstrated that levosimendan significantly improved Cl compared with placebo (SMD 1.16, 95% CI 0.04–2.29), but no statistically significant differences were observed between levosimendan and milrinone, dobutamine, or standard therapy (Figure 3A). Notably, in the SUCRA statistical analysis, milrinone was ranked as the most effective intervention for improving CI (SUCRA 84.3%), with levosimendan (78.6%), dobutamine (35.0%), placebo (33.1%), and standard therapy (19.1%) following in descending order (Table 2). Funnel plot asymmetry indicated the possible presence of publication bias in the analyzed studies (Supplementary Figure S2A).

**Table 2:** Comparison of SUCRA scores for hemodynamic parameters and ICU stay across different treatments.

Control/SUCRA (%)	Dobutamine	LEVO	Milrinone	Placebo	Standard therapies
CI	35	78.6	84.3	33.1	19.1
MAP	89	47.5	3.2	70.4	39.9
CVP	96.7	27.5	45	61.4	19.4
ICU stays	97.5	22.9	22	47.4	60.2

LEVO, levosimendan; CI, cardiac index; MAP, mean systemic artery pressure; CVP, central venous pressure; ICU, intensive care unit.

#### 3.3.2 Central venous pressure

Thirteen trials evaluated the effects of different inotropic agents on CVP (5, 6, 16, 29, 31, 32, 34, 37, 43–46) (Supplementary Figure S1B). Network meta-analysis revealed that milrinone significantly reduced CVP compared with placebo (SMD −0.46, 95% CI −0.83 to −0.09) and further decreased CVP compared with dobutamine (SMD −0.66, 95% CI −1.22 to −0.10). In contrast, levosimendan was associated with a marked increase in CVP compared with milrinone (SMD 0.40, 95% CI 0.05–0.75) (Figure 3B).

SUCRA analysis demonstrated a distinct hierarchy of efficacy for CVP reduction: dobutamine (SUCRA 89.0%) had the highest probability of superior efficacy, followed by placebo (70.4%), levosimendan (47.5%), standard therapy (39.9%), and milrinone (3.2%) (Table 2). Although SUCRA suggested a potential advantage for dobutamine, the network meta-analysis found no statistically significant differences among levosimendan, placebo, and standard therapy (*p* > 0.05). Funnel plot symmetry and Egger's regression test (*p* < 0.05) confirmed minimal publication bias (Supplementary Figure S2B).

#### 3.3.3 Mean systemic artery pressure (MAP)

Seventeen RCTs were included to evaluate the hemodynamic effects of levosimendan vs. other agents on MAP (5, 6, 29, 31, 32, 34, 36–38, 40, 42–46) (Supplementary Figure S1C). The network meta-analysis indicated that both levosimendan (SMD −0.93, 95% CI −1.63 to −0.22) and standard therapy (SMD −1.11, 95% CI −2.18 to −0.05) significantly reduced MAP compared with dobutamine (Figure 3C). No statistically significant differences were observed between levosimendan and other inotropic agents. SUCRA analysis ranked dobutamine highest for MAP preservation (96.4%), followed by placebo (61.4%), milrinone (45.0%), levosimendan (27.5%), and standard therapy (19.4%) (Table 2). Funnel plots showed mild asymmetry, as corroborated by Egger's regression test (Supplementary Figure S2C).

#### 3.3.4 ICU stays

Twenty-one trials were systematically evaluated to assess the effects of levosimendan and other agents on ICU stays in cardiac surgery (5, 7, 8, 10, 12, 16–18, 28, 32, 34, 35, 37–42, 45, 47, 48) (Supplementary Figure S1D). Network meta-analysis revealed that milrinone significantly reduced ICU stays compared with dobutamine (SMD −0.71, 95% CI −1.25 to −0.17) and levosimendan similarly shortened ICU stays compared with dobutamine (SMD −0.68, 95% CI −1.13 to −0.24). In contrast, dobutamine was associated with prolonged ICU stays compared with placebo (SMD 0.58, 95% CI 0.09–1.07) (Figure 3D). SUCRA analysis demonstrated the following hierarchy for ICU stays optimization: dobutamine (97.5%), standard therapy (60.2%), placebo (47.4%), levosimendan (22.9%), and milrinone (22.0%) (Table 2). Funnel plot symmetry (Supplementary Figure S2D) and Egger's regression test confirmed minimal publication bias.

#### 3.3.5 Creatinine

Eight studies assessed the effects of levosimendan on creatinine levels compared with alternative therapies (5, 7, 16, 28, 34, 37, 45, 49) (Supplementary Figure S1E). The network meta-analysis revealed no statistically significant differences in creatinine changes between levosimendan and other inotropic agents (Figure 3E). Funnel plot mild asymmetry indicates potential publication bias, suggesting possible underrepresentation of small-scale studies with neutral or negative findings (Supplementary Figure S2E).

#### 3.3.6 Adverse events and long-term outcomes

Several included trials reported safety outcomes such as hypotension, arrhythmias, and low cardiac output syndrome, as well as short-term mortality (e.g., in-hospital, 30-day, or 90-day death) and rehospitalization. However, the definitions and reporting of adverse events were heterogeneous across studies, and the number of events was relatively low. Overall, levosimendan was not associated with a higher incidence of ventricular or supraventricular arrhythmias compared with dobutamine or milrinone, and episodes of clinically significant hypotension were comparable between groups in most trials (16–18, 34, 39, 41).

With regard to hard clinical endpoints, large multicenter RCTs in high-risk cardiac surgery patients have shown no significant reduction in 30-day or 90-day mortality with perioperative levosimendan compared with placebo (16–18). In our network meta-analysis, the available data on mortality and other long-term outcomes were too sparse and inconsistent to allow a robust quantitative synthesis. Therefore, these endpoints were summarized qualitatively.

## 4 Discussion

Levosimendan was first approved for clinical use in Sweden in 2000 for the hemodynamic stabilization of patients with acutely decompensated chronic congestive heart failure (1). This innovative therapy has received approval in more than 60 countries and is currently undergoing Phase III clinical trials in the United States. Over the past two decades, its clinical applications have expanded substantially, now encompassing a broad range of indications including cardiogenic shock, advanced HF, right ventricular failure, pulmonary hypertension, cardiac surgery, and even non-cardiac conditions such as amyotrophic lateral sclerosis (50).

Levosimendan has generally been well tolerated, with a safety profile that compares favorably to other inotropes, particularly regarding arrhythmia risk and myocardial oxygen demand. Currently, levosimendan is mainly used in the clinical management of acute decompensated heart failure, especially for patients who are unresponsive to conventional inotropes or have renal impairment (15). It is also increasingly applied as perioperative support in high-risk cardiac surgery, where evidence suggests benefits in reducing postoperative complications such as renal injury and shortening mechanical ventilation duration, although its effect on long-term mortality remains uncertain. Recent meta-analyses indicate that intermittent administration of levosimendan may improve cardiac function and symptoms in patients with advanced or chronic heart failure, but its routine use outside selected patient populations is not widely recommended in current guidelines (51). Overall, levosimendan is reserved for specific clinical scenarios, and further large-scale studies are needed to clarify its broader role in heart failure management. Ongoing clinical trials are evaluating the utility of levosimendan across a range of cardiac and non-cardiac conditions, including neurodegenerative diseases such as amyotrophic lateral sclerosis. Its unique capacity to sensitize skeletal muscle fibers to calcium enables the enhancement of submaximal force generation without increasing energy consumption (13, 52).

While early single-center and small-scale studies suggested potential perioperative benefits of levosimendan, recent large multicenter RCTs and meta-analyses have failed to consistently demonstrate its superiority over other inotropic agents. The heterogeneity in study designs and patient populations has contributed to these inconsistent findings, resulting in a lack of unanimous clinical recommendations (13, 15, 53). The optimal timing for levosimendan administration, patient subgroups (e.g., varying HF risk), and dosing regimens remain unclear. Furthermore, there are limited large head-to-head trials directly comparing levosimendan with other inotropic agents. Evidence is also insufficient regarding its use in special populations, such as pediatric or elderly patients and those with multi-organ dysfunction. In addition, further research is needed to clarify the roles of its active metabolites and its interactions with immunological and inflammatory pathways.

In this study, we employed a network meta-analysis to systematically compare the efficacy and safety of levosimendan with other inotropic agents in patients undergoing cardiac surgery, including milrinone, dobutamine, and placebo. By integrating data from 29 randomized controlled trials, we constructed a comprehensive evidence network that allowed for both direct and indirect comparisons across multiple interventions. Unlike traditional pairwise meta-analyses, network meta-analysis enables the simultaneous evaluation of multiple treatments, thereby maximizing the use of available evidence. This approach enhances the precision of effect estimates and provides a more complete picture of the relative benefits and risks of each intervention. To our knowledge, this network meta-analysis provides updated and comprehensive evidence regarding the efficacy of levosimendan compared with other inotropic therapies in cardiac surgery patients. The findings reveal differentiated hemodynamic efficacy and clinical outcomes among these agents, providing critical evidence for personalized therapeutic decision-making. Our network meta-analysis showed that levosimendan increased postoperative cardiac index compared with placebo (SMD 1.16, 95% CI 0.04–2.29) and reduced mean arterial pressure compared with dobutamine (SMD −0.93, 95% CI −1.62 to −0.23). Both levosimendan and milrinone shortened ICU length of stay compared with dobutamine (SMD −0.68, 95% CI −1.13 to −0.24 and SMD −0.71, 95% CI −1.25 to −0.17, respectively). In contrast, no significant differences in creatinine levels were observed among the inotropic regimens. These results indicate that levosimendan has favorable effects on perioperative hemodynamics and ICU stay but a neutral effect on renal function in the available trials.

### 4.1 Hemodynamic results

Previous meta-analyses have found that levosimendan exhibits significant hemodynamic advantages in heart failure patients compared with conventional therapies. Specifically, it reduces pulmonary capillary wedge pressure and systemic venous congestion while markedly increasing CI, reflecting improved ventricular–vascular coupling efficiency and myocardial performance (54–56). The current systematic review identified significant hemodynamic improvements associated with levosimendan therapy, including reduced CVP, increased systolic blood pressure, and urine output (57). However, when compared specifically with milrinone, the analysis revealed no significant cardioprotective advantage for levosimendan (34). Our network meta-analysis extends these findings by showing that levosimendan significantly improved CI compared with placebo (SMD = 1.30), although its efficacy remained secondary to milrinone (SUCRA 84.3% vs. 78.6%). Notably, levosimendan increased CVP compared with milrinone (SMD = 0.40). This suggests that milrinone through a direct inhibition of PDE-3 reduces venous capacitance resistance, optimizing right ventricular preload, whereas levosimendan enhances cardiac function by promoting effective calcium ion binding to troponin C, enhancing contractility while preserving hemodynamic stability.

Levosimendan significantly reduced MAP compared with dobutamine (SMD = −0.93), and dobutamine ranked highest in SUCRA (96.4%). However, dobutamine may induce irreversible myocardial cellular injury through β-adrenergic receptor-mediated oxidative stress and pathological calcium overload, whereas levosimendan opens mitochondrial ATP-sensitive potassium channels in cardiovascular tissues and exerts peripheral vasodilatory and anti-ischemic effects (58, 59). Levosimendan is a novel calcium-sensitizing inotropic agent that augments myocardial contractility by enhancing the myofilament sensitivity to calcium via binding to cardiac troponin C in a calcium-dependent manner without increasing intracellular calcium or levels augmenting myocardial oxygen demand. These pharmacological properties collectively reduce the risks of arrhythmogenesis and ischemia that are typically associated with conventional positive inotropic agents (60). Additionally, levosimendan improves ventricular diastolic function and reduces cardiac stress by its vasodilatory effects. These combined hemodynamic effects synergistically improve cardiac performance, rendering it particularly efficacious in the management of acute decompensated heart failure and hemodynamically unstable states characterized by hypotension (61). Moreover, levosimendan has anti-inflammatory and anti-apoptotic properties, which may contribute to myocardial protection in various clinical settings. Studies have shown that levosimendan and its metabolites decrease circulatory pro-inflammatory cytokines and exhibit anti-apoptotic effects (62). Following administration, only a small fraction of levosimendan is converted into its active metabolite OR-1896 through the intermediate OR-1855. These metabolites, characterized by their extended half-life of approximately 70–80 h, are considered key contributors to the prolonged clinical effects of levosimendan. Importantly, while levosimendan itself can suppress the cytokine-induced upregulation of adhesion molecules and inflammatory mediators in endothelial cells, OR-1855 and OR-1896 mainly demonstrate anti-inflammatory actions by reducing IL-1β-triggered reactive oxygen species (ROS) generation via inhibition of mitogen-activated protein kinase (MAPK) p38, extracellular signal-regulated kinase 1/2 (ERK1/2), and c-Jun N-terminal kinase (JNK) signaling pathways, rather than directly affecting adhesion molecule expression (63).

### 4.2 Safety and tolerability

Across the included RCTs, levosimendan was generally well tolerated in the perioperative setting and showed a safety profile comparable to, and in some respects more favorable than, traditional catecholaminergic inotropes (16–18, 34, 39, 41). Mechanistically, these safety observations are compatible with the pharmacological profile of levosimendan. Postoperative LCOS is typically driven by myocardial stunning, pre-existing ventricular dysfunction, ischemia–reperfusion injury, and systemic inflammation, whereas catecholamine and phosphodiesterase-III inotropes counteract this state at the cost of increased intracellular calcium, myocardial oxygen consumption, and arrhythmogenicity (9, 54, 58, 59). Levosimendan, in contrast, enhances contractility mainly through calcium sensitization of troponin C and activation of mitochondrial and vascular ATP-sensitive potassium channels, thereby supporting ventricular–arterial coupling and organ perfusion with less calcium loading (1, 3, 14, 58–60, 64). In line with this, randomized trials and meta-analyses have not demonstrated a clear or consistent excess of LCOS, clinically significant arrhythmias, or ischemic events in levosimendan-treated patients compared with dobutamine or milrinone, although individual study findings are heterogeneous (14, 15, 24, 34, 39). From a pharmacological perspective, levosimendan is less likely than catecholamine or phosphodiesterase-III inotropes to promote calcium overload-mediated arrhythmias or additional myocardial ischemia, but its vasodilatory properties make hypotension and reflex tachycardia more frequent and clinically relevant adverse effects, particularly in hemodynamically unstable or vasoplegic patients (16–18, 24).

In perioperative RCTs, several studies reported similar or lower rates of ventricular and supraventricular arrhythmias with levosimendan compared with dobutamine or milrinone, despite its potent inotropic effects (16–18, 34, 39). Furthermore, although levosimendan exerted vasodilatory actions and reduced MAP relative to dobutamine, clinically significant hypotension requiring treatment escalation was not consistently increased in the levosimendan arms (16–18, 34, 41). These observations are broadly in keeping with previous meta-analyses in acute and advanced heart failure, which suggest that levosimendan may be associated with a lower risk of arrhythmias and myocardial ischemia than catecholamine-based inotropes, consistent with its calcium-sensitizing rather than calcium-loading mechanism of action (1, 50, 51, 54).

These observations are consistent with previous meta-analyses in acute and advanced heart failure, which have suggested that levosimendan may be associated with a lower risk of arrhythmias and myocardial ischemia than catecholamine-based inotropes, owing to its calcium-sensitizing rather than calcium-loading mechanism of action (1, 50, 51, 54).

### 4.3 Clinical outcomes

Current treatment strategies for acute heart failure predominantly depend on β-adrenergic agonists (dopamine, dobutamine) and catecholamines (epinephrine), which significantly increase myocardial oxygen consumption and are associated with prolonged ICU stays (57). Previous study suggested that combining dobutamine with levosimendan, compared with dobutamine with milrinone, was associated with reduced postoperative complication rates and consequently shorter ICU stays in cardiac surgery patients with a low preoperative ejection fraction (34). Our findings corroborate previous studies demonstrating that levosimendan reduces ICU length of stay in cardiac surgery patients (41, 65). Compared with the calcium sensitizer levosimendan and PDE inhibitor milrinone, dobutamine may significantly prolong ICU stay through multiple mechanisms: (1) its combined positive inotropic and chronotropic effects exacerbate myocardial oxygen consumption, further aggravating ischemia; (2) potent β-adrenergic stimulation increases arrhythmogenic risk; and (3) patients requiring postoperative dobutamine infusion likely represent a cohort with greater baseline critical illness severity.

The renal protective effects of levosimendan warrant particular attention. Given that acute kidney injury constitutes a major driver of morbidity and mortality postcardiac surgery, strategies to preserve renal function are critical (66, 67). Recent studies investigated the effect of dobutamine and levosimendan on glomerular filtration rate and reported that levosimendan significantly improved renal filtration rate (5, 68, 69). Potential underlying mechanisms include systemic hemodynamic optimization through enhanced cardiac output and reduced central venous pressure, improved renal hemodynamics, and direct cellular effects including anti-inflammatory and anti-apoptotic activities (64, 70). Our study found no significant difference in postoperative serum creatinine levels between levosimendan and other control treatments, indicating that levosimendan and the other inotropic agents are equally safe for renal function in patients undergoing cardiac surgery.

Despite these hemodynamic and ICU-related benefits, the included trials did not demonstrate a consistent reduction in in-hospital, 30-day, or 90-day mortality with levosimendan compared with placebo, dobutamine, or milrinone. The decreases in ICU stay were moderate in magnitude and were mainly observed in comparison with dobutamine, whereas overall hospital length of stay and longer-term outcomes were either rarely reported or showed no clear advantage. Thus, the available evidence suggests that levosimendan improves short-term hemodynamic profiles, but its impact on major clinical endpoints, particularly mortality, remains uncertain.

While levosimendan demonstrated significant improvements in hemodynamic parameters compared with placebo, our analysis did not reveal clear superiority over other inotropic agents such as milrinone or dobutamine. These findings indicate that levosimendan may serve as a viable alternative or adjunct to conventional inotropes, particularly in perioperative cardiac surgery patients with compromised renal function or those who may not tolerate adrenergic stimulation. Given these nuanced findings, clinical exploration of prophylactic use, early administration, and combination therapy with other inotropic agents may be considered in selected high-risk perioperative patients, although current evidence remains insufficient to recommend routine use over established agents.

To further clarify the role of levosimendan in this context, large-scale, prospective, randomized controlled trials are urgently needed. These studies should focus not only on comparative efficacy and safety but also on optimal dosing strategies and timing of administration, including the potential benefits of prophylactic use in selected high-risk populations. Additionally, future research should incorporate long-term clinical outcomes and patient-centered endpoints to better inform clinical decision-making.

## 5 Limitations

This study had some limitations that should be addressed. Firstly, the network meta-analysis incorporated clinically heterogeneous populations encompassing various cardiac surgery types (including coronary artery bypass grafting, valve procedures, congenital heart disease, and combined surgeries) with broad age distributions (neonates to older adults), baseline ventricular function, and drug dosing regimens. Because individual patient-level data were not available and many trials did not report stratified results, we were unable to perform predefined subgroup analyses by age, baseline disease severity [e.g., left ventricular ejection fraction (LVEF), presence of pulmonary hypertension, or chronic kidney disease], or type of cardiac surgery. The absence of these subgroup analyses may have introduced substantial clinical heterogeneity and limited the ability to tailor our findings to specific patient groups. Secondly, only a minority of the included studies used standard therapy as the comparator, whereas most employed placebo controls, which may overestimate treatment effects and underrepresent real-world clinical decision contexts. Furthermore, another limitation of this analysis is the wide confidence intervals observed for some meta-analytic results, reflecting imprecision in the data. This imprecision may be due to the limited number of studies or small sample sizes included in these particular comparisons, which might partly explain the observed asymmetry in the funnel plots. These limitations highlight the need for future research incorporating standardized protocols, active-comparator designs, future larger-scale studies, and rigorous subgroup analyses to validate our findings.

## 6 Conclusion

In conclusion, this network meta-analysis suggests that perioperative levosimendan provides favorable effects on hemodynamics and ICU length of stay in cardiac surgery patients, while its impact on renal function and long-term mortality remains uncertain. Among the various inotropic agents, levosimendan demonstrates substantial benefits in enhancing cardiac function and hemodynamic stability while reducing ICU length of stay. Owing to its unique mechanism of action and favorable safety profile, levosimendan represents a promising therapeutic option for managing acute postoperative heart failure.

## Data Availability

The datasets presented in this study can be found in online repositories. The names of the repository/repositories and accession number(s) can be found in the article/Supplementary Material.
