# Supplementary material for: Effect of levosimendan treatment in cardiac surgery: a network meta-analysis of randomized controlled trials
Source: Front Cardiovasc Med. 2026 Feb 11;12:1673410. doi: 10.3389/fcvm.2025.1673410 (PMC12932496; doi:10.3389/fcvm.2025.1673410)
Supplement: Supplementary file 1 [file Datasheet1.docx]

*Supplementary Materials*

**Effect of Levosimendan Treatment in Cardiac surgery: A Network Meta-Analysis of Randomized Controlled Trials**

Binlu Zhu, Wanling Zhao, Yifei Li*

Department of Pediatrics, West China Second University Hospital, Sichuan University, Chengdu, Sichuan 610041, China.

* Corresponding author: Yifei Li

Dept. of Pediatrics, West China Second University Hospital, Sichuan University

No. 20, 3rd section, South Renmin Road

Chengdu, 610041, China

Email: [liyfwcsh@scu.edu.cn](mailto:liyfwcsh@scu.edu.cn)


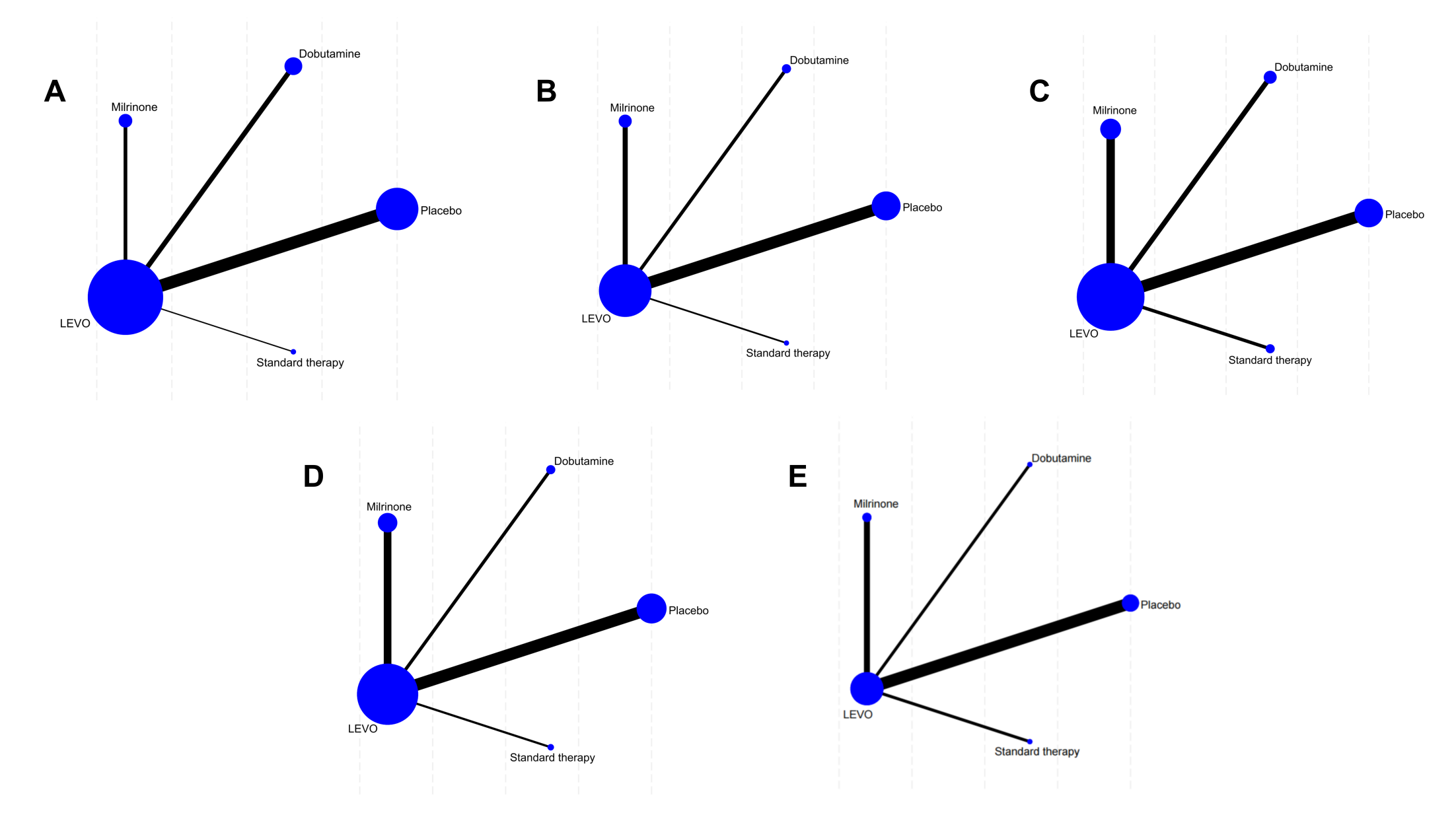


**Supplementary Figure 1.** Network plot for included therapies of CI (A), CVP (B), MAP (C), ICU stays (D) and creatinine (E).

Levosimendan (LEVO); cardiac index (CI), central venous pressure (CVP), mean systemic artery pressure (MAP); intensive care unit (ICU)

**
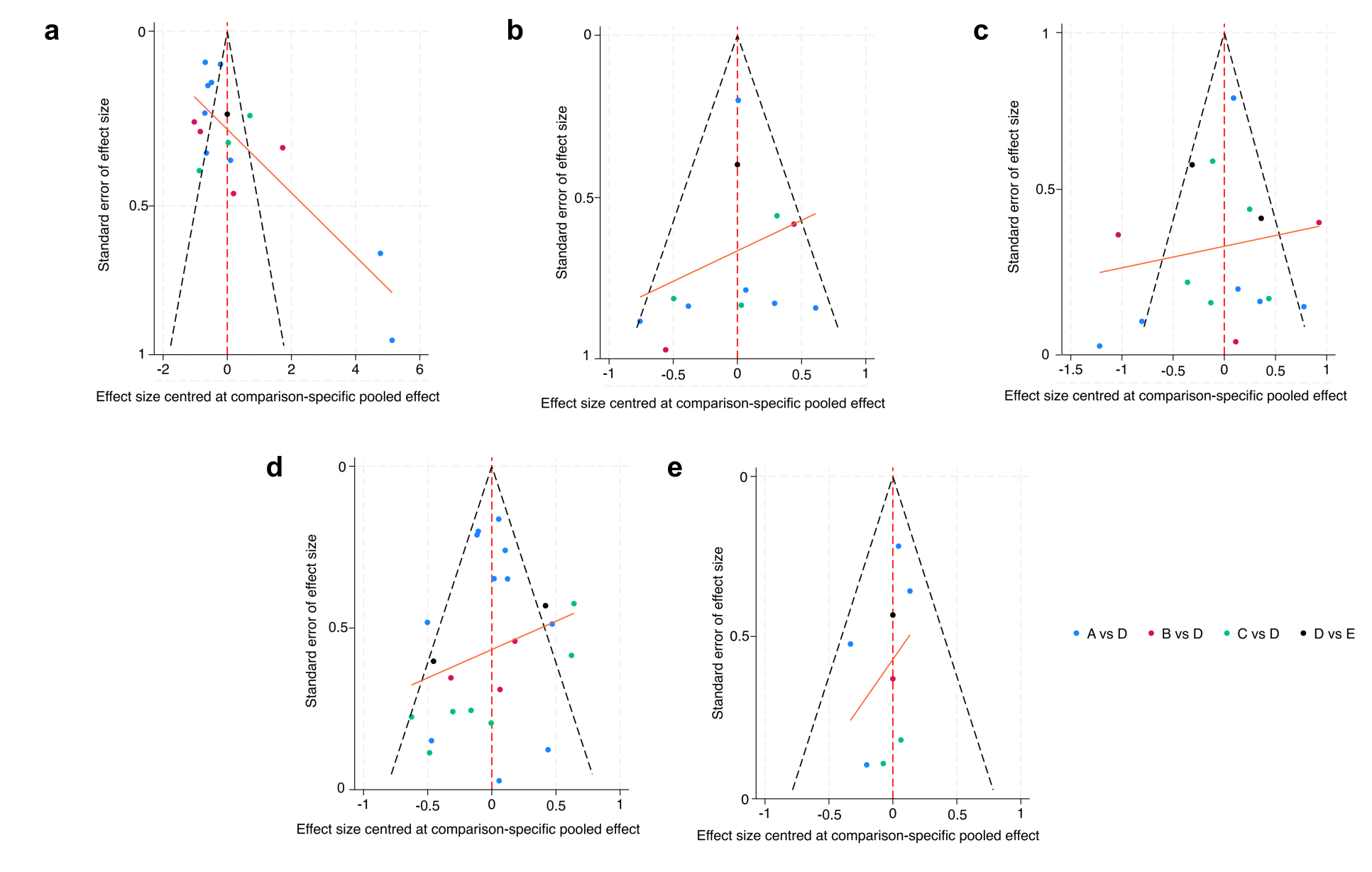
**

**Supplementary Figure 2.** Funnel plot with Deek’s test for diagnostic analysis of CI (a), CVP (b), MAP (c), ICU stays (d) and creatinine (e).

A: placebo; B: dobutamine; C: milrinone; D: levosimendan; E: standard inotropic therapies

cardiac index (CI), central venous pressure (CVP), mean systemic artery pressure (MAP); intensive care unit (ICU)
